# Literature review of imaging, pathological diagnosis, and outcomes of metachronous lung and pancreatic metastasis of cecal cancer

**DOI:** 10.1186/s12957-022-02797-7

**Published:** 2022-10-17

**Authors:** Xiang Wu, Shuping Zhou, Xinhua Zhou, Xiao Xu, Luoluo Wang, Yi Ruan, Jun Lu, Haili Li, Hongfeng Xu, Xinyi Ma, Hong Li

**Affiliations:** 1 Department of Hepatobiliary and Pancreatic Surgery, Affiliated Li Huili Hospital, Ningbo University School of Medicine, Ningbo, 315000 China; 2 Ningbo Municipal Health Vocational and Technical College, Ningbo, 315100 China; 3 Department of Hepatobiliary and Pancreatic Surgery, Affiliated Hangzhou First People’s Hospital, Zhejiang University School of Medicine, Hangzhou, 310006 China; 4 Department of Diagnosis, Ningbo Diagnostic Pathology Center, Ningbo, 315000 China; 5 Department of General Surgery, The Third People’s Hospital Health Care Group of Cixi, Ningbo, 315300 China; 6 Department of Pathology, Hangzhou DIAN Medical Laboratory, Hangzhou, 310000 China

**Keywords:** Colon cancer, Pancreatic metastases, Lung metastases, Immunohistochemistry, NGS, Treatment

## Abstract

**Background:**

Pancreatic metastasis from colorectal cancer is extremely rare. Here, we report a case of colorectal cancer with lung and pancreatic metastasis and analyze the histopathology, immunohistochemistry, and next-generation sequencing (NGS) to generate a differential diagnosis and treatment of metastatic colon cancer.

**Case presentation:**

AC1

A 78-year-old man was admitted because of a recently elevated carcinoembryonic antigen. This patient had undergone laparoscopic right hemicolectomy for cecal cancer IIA (T3N0M0) 5 years before admission, and thoracoscopic left upper lung wedge resection for primary colon cancer lung metastasis 2 years before admission. At that time, the patient was thought to have pancreatic metastasis from colon cancer. He underwent laparoscopic distal pancreatectomy (combined with splenectomy). Postoperative pathology revealed colon cancer metastasis. We performed NGS on tumor samples at three loci and found colon cancer's most common oncogenic driver genes (KRAS, APC, and TP53). One month after surgery, the patient was given capecitabine for six cycles of chemotherapy. At present, no high adverse reactions have been reported.

**Discussion:**

For patients with pancreatic space-occupying, such as a previous history of colorectal cancer, and recent carcinoembryonic antigen elevation, we should highly suspect pancreatic metastatic colorectal cancer. NGS is an essential auxiliary for identifying metastatic tumors. Surgery combined with postoperative chemotherapy is an effective treatment.

## Introduction

The common colorectal cancer (CRC) metastasis sites include the surrounding lymph nodes, liver, and lung [1]. Metastasis in the pancreas is rare, accounting for 2% of all pancreatic malignancies [2–5]. There are no evident clinical symptoms in the early stage of pancreatic metastasis; the most common presenting symptoms are abdominal pain, abdominal distension, jaundice, weight loss, loss of appetite, and abnormal blood glucose. Pancreatic space-occupying lesions are found on physical examination and imaging, and most cases are discovered in the late stage [6, 7]. After resection, the diagnosis often requires puncture cytological pathology or pathological examination [8].

Pancreatic cancer is usually treated with chemotherapy or surgery combined with chemotherapy [9]. Preoperative diagnosis of pancreatic cancer relies on tumor indicators such as recent carcinoembryonic antigen elevation, CA-199, and computed tomography (CT). CT imaging facilitates the detection of lesions and the assessment of peritumor invasion and metastasis. The final diagnosis relies on histopathology and immunohistochemistry, and NGS can improve the differential diagnosis of metastatic tumors. We report the diagnosis and treatment of a patient with metachronous lung and pancreatic metastasis of cecal carcinoma.

## Case presentation

A 78-year-old man presented with elevated carcinoembryonic antigen (CEA) levels. Abdominal CT revealed a pancreatic tumor, and he was transferred to our hospital. The patient had a history of laparoscopic right hemicolectomy for cecal cancer IIA (T3N0M0) 5 years before admission. He presented with nausea, vomiting, numbness of fingers, and other complaints after two rounds of oxaliplatin combined with capecitabine. The patient refused oxaliplatin and was switched to capecitabine alone for four cycles. Two years before admission, he underwent thoracoscopic left upper lung wedge resection due to lung metastasis of his primary colon cancer. Postoperative pathology and immunohistochemistry suggested that the primary tumor was colon cancer. Oral drug therapy was not continued after surgery and six cycles of capecitabine chemotherapy.

**Fig. 1:**
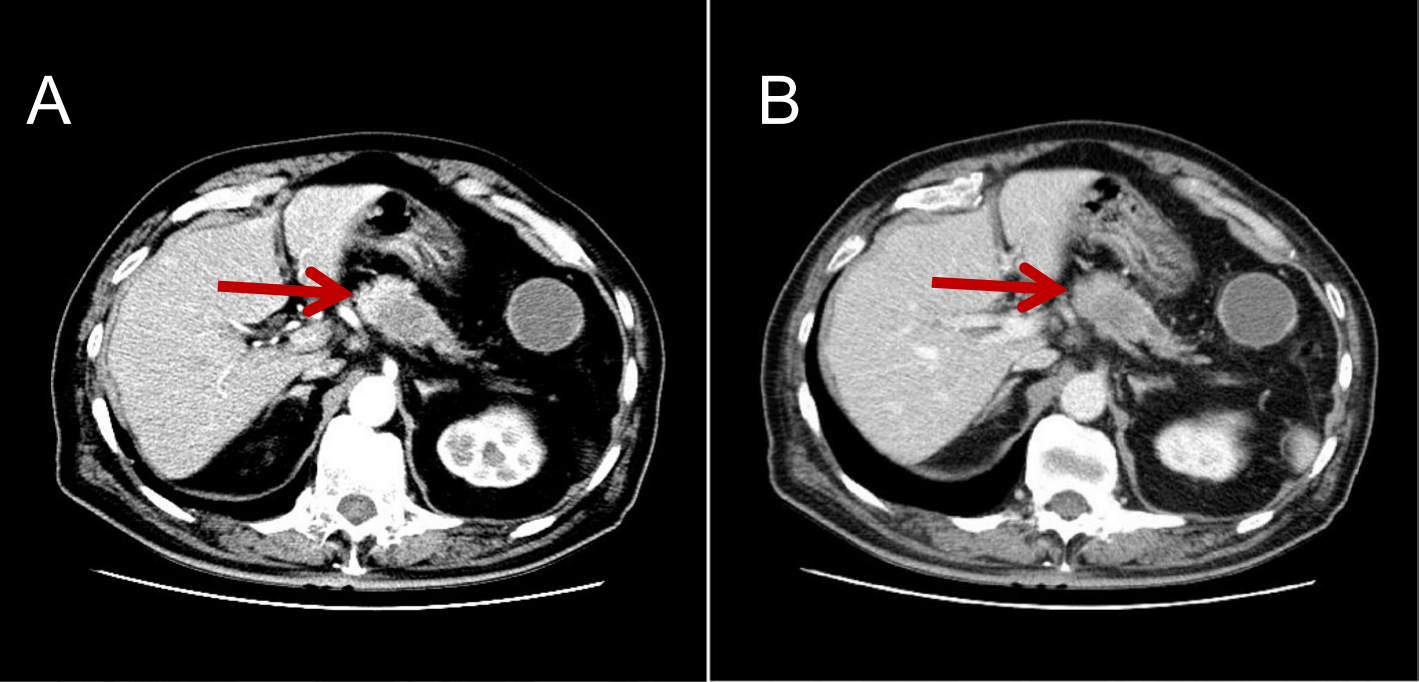
Abdominal CT scan showing a 5.5 × 2.8 × 2 cm mass in the pancreas (indicated by the arrow). **A** Marginal enhancement of the arterial area. **B** No enhancement in the venous area

Shortly before admission, the patient’s CEA increased to 9.2 ng/ml (normal 0–5 ng/ml), CA-199: 23.8 U/ml (normal 0–37 U/ml), and AFP: 4.3 ng/ml (normal 0–8.8 ng/ml). No abnormalities in routine complete blood count, liver enzymes, or kidney function were found. A CT at our hospital revealed a pancreatic tumor measuring 5.5 × 2.8 × 2 cm, suggesting a malignancy (Fig. 1A, B).

**Fig. 2:**
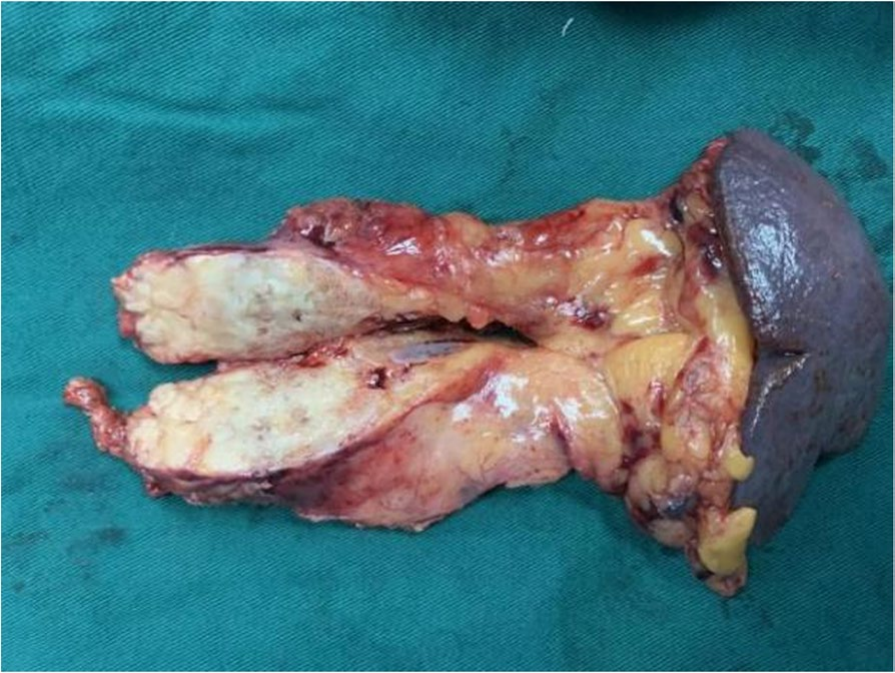
Postoperative surgical specimen: pancreatic tail and spleen

Because the patient had a previous history of colon cancer and recent CEA elevation, we suspected pancreatic metastatic colon cancer. Because he was found to have no contraindications for surgery, the patient underwent laparoscopic pancreatectomy (combined with splenectomy). No significant adhesions were found in the peripancreatic tissue. The pancreatic body size was approximately 5 × 3 × 2 cm (Fig. 2). The intraoperative frozen sections revealed negative margins. Intraoperative blood loss was 100 ml, and there was no transfusion.

**Fig. 3:**
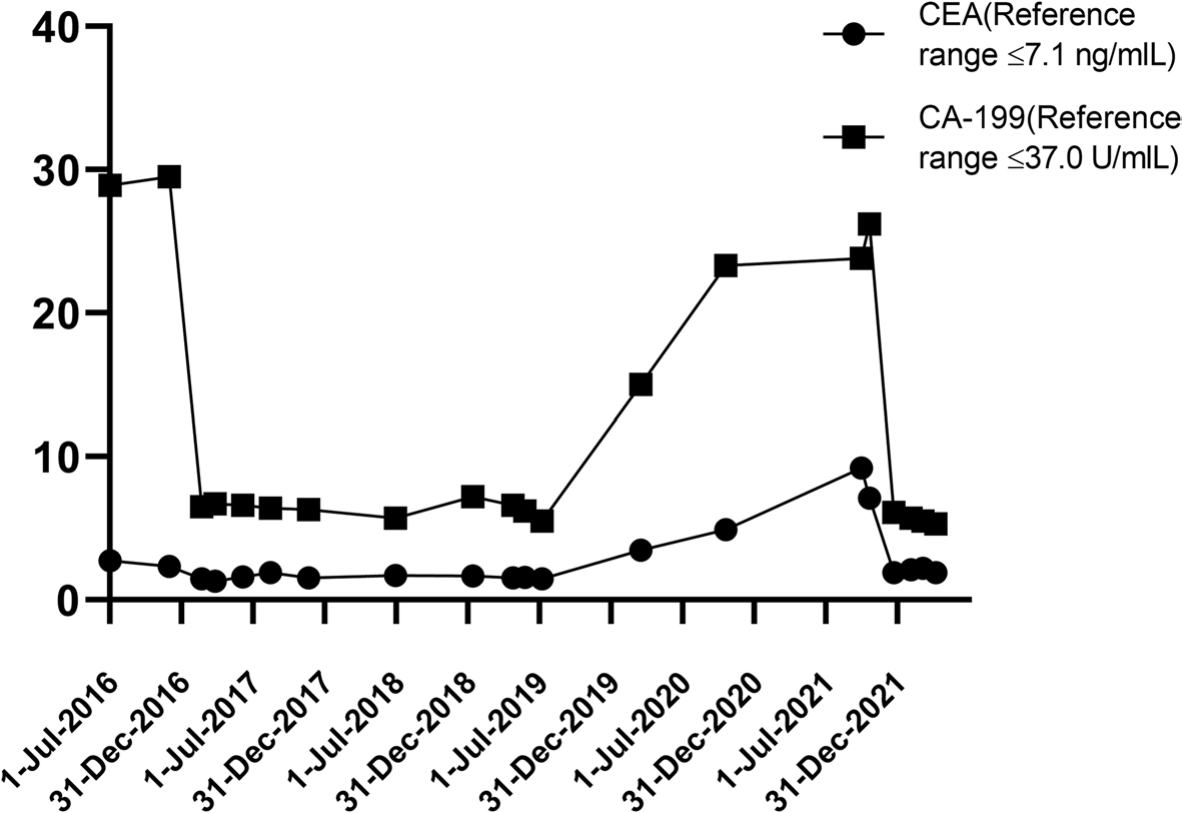
Changes in the disease CEA and CA-199 in this patient

Postoperatively, there was no pancreatic fistula, abdominal infection, or bleeding. Ten days after the operation, the patient was discharged after removing the drainage tube. One month after the operation, the patient returned to the hospital for an examination. He had no complaints of discomfort. A complete blood count, liver enzymes, and kidney functions were normal. The tumor marker CEA had returned to normal. The postoperative specimen is shown in Fig. 3.

**Fig. 4:**
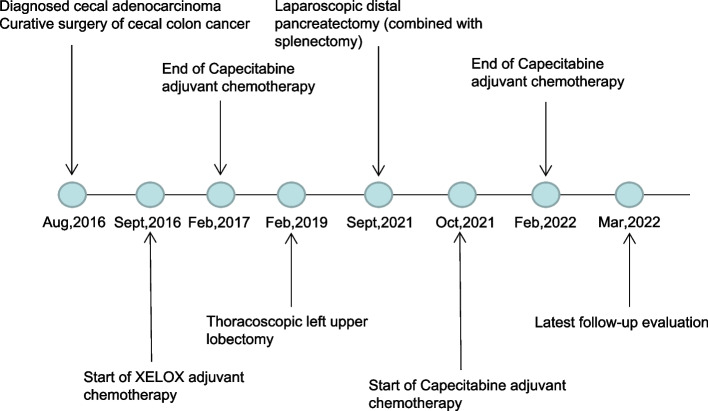
Disease treatment process of the case described in this patient

Histopathology and immunohistochemistry revealed pancreatic adenocarcinoma, CDX-2 (+), villin (+), CD20 (+), CK7 (−), CK19 (+), Ki-67 (+) 60%, and Satb2 (+). Our conjecture was confirmed. We performed mutation analysis on 520 cancer-related genes in tumor samples at three loci (Nanjing Shihe gene Bio-Technology Co., Ltd, China). As shown in Table 1, ten appeared in three samples simultaneously among the 21 mutant genes detected. Because the patient refused oxaliplatin, we continued treatment with oral capecitabine. The patient received a total of six cycles of oral capecitabine. No significant adverse drug reactions were observed (Fig. 4).

**Table 1:** Targeted next-generation sequencing of 520 cancer-related genes from the primary sigmoid cancer, lung metastasis, and pancreatic resection

Mutated gene and impact on protein sequence	Cecal tumor	Lung tumor	Pancreatic tumor
*KRAS* p.G12D	+	+	+
*APC* p.K1310Rfs*11	+	+	+
*APC* p.H1490Ifs*17	+	+	+
*TP53* c.560-1G>A	+	+	+
*TP53* p.T81Nfs*68	+	+	+
*TP53* p.R273H	+	+	+
*FBXW7* p.E573*	+	+	+
*FBXW7* p.G663R	+	+	+
*PTEN* p.T319fs	+	+	+
*PIK3CA* p.E545K	+	+	+
*PIK3CA* p.E542K	+		
ERCC1 p·N118N	+		
*ERCC5* p.T106M	+		
CDKMN2A p.R80*			+
*LRP1B* p.A3643D	+		
*LRP1B* p.P1191T		+	
XRCC1 p·Q399R	+		
*TSHR* p.D487N			+
*ASXL1* p.G668S			+
*FGF3* p.A87S		+	
*EZH2* p.D731fs		+	

+ positive, *KRAS* KRAS proto-oncogene, GTPase; *APC* APC regulator of WNT signaling pathway, *TP53* tumor protein p53, *FBXW7* F-box and WD repeat domain containing 7, *PTEN* phosphatase and tensin homolog, *ERCC1* excision repair cross-complementing group 1, *ERCC5* excision repair cross-complementing rodent repair deficiency, complementation group 5, *CDKMN2A* cyclin-dependent kinase inhibitor 2A, *LRP1B* LDL receptor-related protein 1B, *PIK3CA* phosphatidylinositol-4,5-bisphosphate 3-kinase catalytic subunit alpha, *XRCC1* X-ray repair complementing defective repair in Chinese hamster cells 1, *TSHR* thyroid-stimulating hormone receptor, *FGF3* fibroblast growth factor 3

## Discussion and conclusions

Common sites of CRC metastasis are the surrounding lymph nodes, liver, and lung [1]. Pancreatic metastasis caused by CRC is rare [2–5], although there are some reports [10, 11]. About 80% of isolated pancreatic metastases originate from renal tumors [12]. Metastatic pancreatic cancer accounts for about 2% of all pancreatic tumors [3].

We indexed CRC, pancreas, and metastasis in PubMed from 2010 to 2021 and identified 16 cases (Table 2) [10, 13–27]. There were nine males and seven females, with an average age of 64.8 ± 10.3 years, of which 13 cases were metachronous metastasis and three were simultaneous metastasis. Nine primary tumors originated from the rectum, four from the sigmoid colon, two from the ascending colon, and one from the cecum. The median interval from the first colon tumor resection to the diagnosis of metastatic pancreatic cancer was 50.5 + 28.8 months, excluding three simultaneous metastases. There were 13 cases of metachronous metastasis (81.2%), of which only one received chemotherapy. The final survival was not recorded (Table 2); however, the overall survival with metastatic pancreatic cancer who underwent surgery was the shortest at 17 months. The longest was 64.8 months, and the average survival was 39.6 ± 18.7 months.

**Table 2:** Characteristics of patients who developed pancreatic metastasis from colorectal cancer

No.	First author/year	Age	Sex	Time to pancreas metastasis (mo)	Synchronous (Sy)	Metachronous (Met)	Primary tumor site	Pancreatic site	Pancreatic surgery	Overall survival months (mo)
1.	Yang J/2021 [10]	59	M	50	-	1	Sigmoid	Head	PD	NA
2.	Yagi Y/2020 [13]	44	F	70	-	1	Sigmoid	Tail	DP	NA
3.	Tokuyama S/2016 [14]	50	F	55	-	1	Sigmoid	Tail	DP	40
4.	Tani R/2019 [15]	70	M	64.8	-	1	Rectal	Head	PD	64.8
5.	Hirano M/2021 [16]	50	M	18	-	1	Sigmoid	Tail	DP	NA
6.	Sano I/2017 [17]	77	F	36	-	1	Rectal	Tail	DP	17
7.	Numata K/2020 [18]	68	F	42	-	1	Rectal	Tail	DP	NA
8.	Sakai K/2019 [19]	69	M	80	-	1	Rectal	Tail	DP	NA
9.	Olesinski T/2019 [20]	69	F	52	-	1	Rectal	Tail	DP + splenectomy	49
10.	Saito M/2019 [21]	71	M	120	-	1	Rectal	Tail	DP	NA
11.	Kurihara S/2019 [22]	67	M	21	-	1	Colon ascendens	Head	PD	NA
12.	Li Destri G/2014 [23]	68	M	0	1	-	Rectal	Head	PD + total colectomy	NA
13.	Ohtsubo K/2013 [24]	77	M	0	1	-	Cecal	Head	--	NA
14.	Tamagawa H/2012 [25]	68	F	0	1	-	Rectal	Tail	Colectomy for rectal carcinoma + DP	NA
15.	Lee CW/2010 [26]	76	F	24	-	1	Rectal	Tail	DP	NA
16.	Lasithiotakis K/2010 [27]	53	M	24	-	1	Colon ascendens	Head	PD	27

*PD* pancreaticoduodenectomy, *DP* distal pancreatectomy, *NA* not available

Pancreatic cancer is often occult because of its relatively low incidence. Pancreatic lesions are often found on physical examination. Signs and symptoms include abdominal pain and distention, jaundice, weight loss, loss of appetite, and abnormal blood glucose. Pancreatic metastases are as challenging to identify as primary pancreatic cancer. Most cases are discovered late [6, 7, 28]. Diagnosis often requires biopsy cytology or intraoperative and post-resection pathological examinations [8].

In the present case, our preoperative diagnosis depended on CT and tumor markers, while the final diagnosis depended on postoperative histopathology and immunohistochemistry. The preoperative enhanced CT revealed a slightly low-density shadow in the pancreas and was thought to represent metastatic pancreatic cancer. This disease should be differentiated from pancreatic cancer, and CT often shows localized masses with low or low mixed density, lack of blood supply, dilation of the pancreatic duct, and adjacent vascular invasion [28]. The second differential diagnosis is a solid pseudopapillary tumor of the pancreas; on imaging, the density of these solid tumors is uneven, composed of a mixture of solid and cystic components. On a plain CT scan, the solid component is hypodense or isodensity, while the cystic component is liquid low-density shadow; on the enhanced scan, the solid component is mildly enhanced in the arterial phase and moderately heterogeneously enhanced in the venous and delayed phases. The cystic component does not appear on the enhanced scan [29]. The third differential diagnosis is non-functional pancreatic neuroendocrine tumors, most of which are asymptomatic. CT often reveals large tumors with uneven tumor density, accompanied by calcified nodules or liquefaction and necrosis [30].

Our conjecture was confirmed by postoperative pathological histological examination and immunohistochemistry. Pathology and immunohistochemistry were consistent with adenocarcinoma, intestinal cancer metastasis, negative intravascular tumor thrombus, negative nerve invasion, and absence of lymph node metastasis in ten lymph nodes around the pancreas. Histological markers were as follows: CDX-2 (+), villin (+), CK20 (+), CK7 (−), CK19 (+), Ki-67 (+) 60%, and SATB2 (+).

**Fig. 5:**
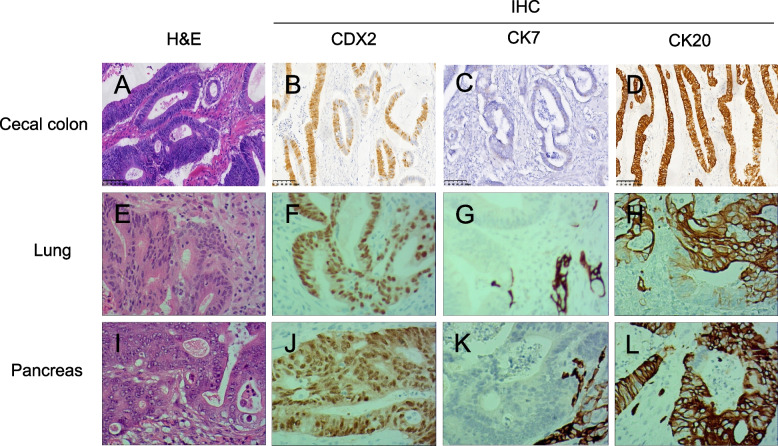
Representative results of hematoxylin and eosin and immunohistochemical staining of primary and metastatic lesion specimens. **A**–**L** In primary cecal adenocarcinomas, H & E staining (**A**) and IHC CDX2 + (**B**), CK7 (−) (**C**), and CK20 + (**D**); similar to the IHC findings in CDX2 + (**F**), CK7− (**G**), and CK20 + (**H**) lung metastasis (**E**–**H**). In addition, pancreatic metastasis (**I**–**L**) also showed a biomarker expression profile consistent with the tissue of cecal cancer origin. H & E, hematoxylin and Shuhong; IHC, immunohistochemistry (original magnification of ×200)

The pathology and immunohistochemistry of the colon provided by the patient’s family indicated that the differentiated adenocarcinoma in the ileocecal part was mucinous adenocarcinoma and ulcerative. Other markers were as follows: P-gp (−), ToPoII (+), MLH1 (+), MSH2 (+), MSH6 (+), PMS2 (+), GST-π (+), CEA (+), CD56 (−), CDX-2 (+), and CK-20 (+). The pathological and immunohistochemical indications of lung metastases were as follows: (left upper lobe) invasive or metastatic adenocarcinoma was considered lung metastasis of CRC, measuring 1.8 cm × 1.8 cm × 1.5 cm. CK20 (+), CK7 (−), TTF-1 (−), napsinA (−), CDX-2 (+), and villin (+). Colon-derived immune markers are CK20, CDX2, and villin, while CK7 is derived from the lung or pancreas and is not expressed in gastrointestinal tumors. CK20 and CDX2 are primarily expressed in gastrointestinal tumors and rarely in pancreatic cancer but lack specificity [31–33]. Our histopathological examinations revealed that the origins of lung cancer and pancreatic cancer were both from the colon (Fig. 5). We analyzed the gene mutation spectrum of three tumor sites. We found that CRC oncogenes appeared at all three sites (KRAS, APC, and TP53) [34], suggesting a common histopathological origin. It is not clear how colon cancer metastasizes to the pancreas. Four mechanisms of metastasis are linked with CRC, specifically, lymphatic spread, direct extension, hematogenous spread, and planting spread. Because the lymph nodes near the pancreas lesion were negative, cancer cells from the colon may have traveled to the pancreas through the bloodstream, which was presumed to be the most probable pathway.

Pancreatic metastasis cancer is primarily treated with chemotherapy and surgery [35, 36]. In Hung’s retrospective analysis of 329 secondary pancreatic tumor resection cases, only 17 cases of pancreatic metastases after colon cancer were found. The most extended interval of postoperative pancreatic metastases was 287 months, the 5-year survival was 24.6%, and the 5-year median survival was 24.0% [37]. Blanco-Fernández et al. found that surgery for pancreatic metastases from renal cell carcinoma improved survival [38]. Chikhladze et al. studied surgical and non-surgical treatment of pancreatic metastases and found that surgery improved survival [39]. Surgical resection of transferred liver metastases confers benefits in colon cancer liver metastasis studies [40, 41]. Because there are few cases of colon cancer with pancreatic metastasis, there is no unified treatment standard. Surgery combined with postoperative chemotherapy is considered effective when comparing pancreatic metastases in other sites. The chemotherapy standard for colon cancer is oxaliplatin combined with capecitabine, and the second option is capecitabine single-drug chemotherapy [42]. We continued capecitabine as single-drug chemotherapy because the patient refused oxaliplatin intravenous chemotherapy. As of this report, the patient had recovered well, the CEA decreased to normal, and no tumor recurrence was observed on the postoperative review.

In conclusion, pancreatic metastasis from colon cancer is rare and may be misdiagnosed as primary pancreatic cancer. For space-occupying lesions of the pancreas, such as those with a previous history of CRC and a recent CEA elevation, metastasis of CRC to the pancreas should be suspected. We believe that a comprehensive NGS analysis of metastatic tumors can improve the differential diagnosis of metastatic tumors. As the case is rarely reported worldwide, there is a lack of consensus regarding treatment; however, surgery combined with postoperative chemotherapy is effective.

## Data Availability

The datasets used and/or analyzed during the current study are available from the corresponding author on reasonable request.
